# High-precision ^14^C and ^40^Ar/^39^Ar dating of the Campanian Ignimbrite (Y-5) reconciles the time-scales of climatic-cultural processes at 40 ka

**DOI:** 10.1038/srep45940

**Published:** 2017-04-06

**Authors:** Biagio Giaccio, Irka Hajdas, Roberto Isaia, Alan Deino, Sebastien Nomade

**Affiliations:** 1 CNR - Istituto di Geologia Ambientale e Geoingegneria (IGAG), Via Salaria km 29.300, 00015 Monterotondo, Rome, Italy; 2 Laboratory of Ion Beam Physics, ETH, Otto-Stern-Weg 5, 8093 Zürich, Switzerland; 3 Istituto Nazionale di Geofisica e Vulcanologia (INGV), Sezione di Napoli, Osservatorio Vesuviano, Via Diocleziano 328, 80124 Naples, Italy; 4 Berkeley Geochronology Center, 2455 Ridge Road, Berkeley, California 94709, USA; 5 Laboratoire des Sciences du Climat et de L’Environnement (IPSL-CEA-CNRS-UVSQ) et Université de Paris-Saclay, Domaine du CNRS Bât. 12, Avenue de la Terrasse, 91198 Gif sur Yvette, France

## Abstract

The Late Pleistocene Campanian Ignimbrite (CI) super-eruption (Southern Italy) is the largest known volcanic event in the Mediterranean area. The CI tephra is widely dispersed through western Eurasia and occurs in close stratigraphic association with significant palaeoclimatic and Palaeolithic cultural events. Here we present new high-precision ^14^C (34.29 ± 0.09 ^14^C kyr BP, 1σ) and ^40^Ar/^39^Ar (39.85 ± 0.14 ka, 95% confidence level) dating results for the age of the CI eruption, which substantially improve upon or augment previous age determinations and permit fuller exploitation of the chronological potential of the CI tephra marker. These results provide a robust pair of ^14^C and ^40^Ar/^39^Ar ages for refining both the radiocarbon calibration curve and the Late Pleistocene time-scale at ca. 40 ka. In addition, these new age constraints provide compelling chronological evidence for the significance of the combined influence of the CI eruption and Heinrich Event 4 on European climate and potentially evolutionary processes of the Early Upper Palaeolithic.

High-precision chronologies and robust correlations between stratigraphic records are of paramount importance for establishing the temporal sequence and relationship between natural events and ultimately for evaluating evolutionary processes. In this framework, the study of distal volcanic ash – ejected into atmosphere during large explosive eruptions and near-simultaneously deposited in diverse sedimentary environments up to thousands of kilometres from the source – has in the last several decades seen increased attention (e.g. ref. 1). Volcanic ash horizons can serve as powerful tools for synchronising natural archives and for transferring information from one stratigraphic record to another, independent of the uncertainties inherent in the comparison of absolute ages, thus enabling assessment of the relative temporal relationships and potential causal connections between disparate processes and events.

Full realization of the potential of a marker tephra is a function of: (*i*) unequivocal correlations from site-to-site based on a host of parameters such as lithology, geochemistry, paleomagnetism, and approximate age; (*ii*) accurate knowledge of its dispersal area, and (*iii*) detailed assessment of stratigraphic events and processes in the sections where the tephra is found. In the framework of western Eurasian tephrostratigraphy, the Campanian Ignimbrite (CI, southern Italy, circa 40 ka^2^), the unique European super-eruption (e.g. ref. 3), is certainly the most widespread (Fig. 1a and b) and relevant tephra marker. Thunnel *et al*.^4^ first recognized the large dispersal of the CI by correlating on-land outcrops to the widespread Y-5 marine layer. Interest in the eruption in the last decade has included recognition of the CI horizon in distal localities (e.g. refs 5, 6, 7, 8, 9, 10, 11, 12), investigation of the geochemical glass composition (e.g. refs 12 and 13), physical volcanology of proximal Plinian deposits (e.g. ref. 14) as well as distal ash (e.g. refs 12 and 15), modelling of eruption dynamics and tephra dispersal (e.g. ref. 3), relationship of the stratigraphic position of the CI tephra relative to palaeoclimatic (e.g. refs 16 and 17) and palaeomagnetic (e.g. ref. 18) events and Palaeolithic cultural entities^5^,^6^,^7^,^8^, the potential impact of the eruption on climate and human ecosystems (e.g. refs 7, 8, 19, 20, 21, 22), and new and improved ^14^C chronology in sections that include the CI tephra^23^,^24^,^25^.

The age and stratigraphic position of the CI are important elements to the investigation of many chronostratigraphic problems in European volcanology, palaeoclimatology and archaeology. Yet despite concerted attempts, the absolute age of the CI eruption is not well constrained. For example, the current ^14^C age for CI relies on the dating of organic samples that are analytically not fully reliable or not directly tied temporally and stratigraphically to the CI eruption (e.g. refs 22 and 23), thus limiting its tephrochronological potential. In regards to ^40^Ar/^39^Ar dating of the eruption, limitations in analytical design and knowledge of the age of standards and decay constants have hampered efforts to achieve the necessary precision and accuracy. Fortunately, recent advancements in machine sensitivity and evaluations of standards and decay constants (e.g. refs 26, 27, 28, 29, 30, 31, 32) now provide the opportunity to improve and reassess the ^40^Ar/^39^Ar age of CI^2^,^33^.

With the aim of fully exploiting the potential of the CI tephra marker, here we present the first direct radiocarbon age for the CI obtained using accepted modern practices, from multiple ^14^C analyses of an exceptional large charred tree branch embedded in the lithified Yellow Tuff facies of the CI pyroclastic flow deposits (Fig. 1c), as well as new ^40^Ar/^39^Ar dating results for the CI. Results are discussed in terms of implications for the radiocarbon calibration, late Pleistocene time-scale, and palaeoecologic and Early Palaeolithic cultural change in western Eurasia.

## Results

### Campanian Ignimbrite ^14^C age

**Figure 1:**
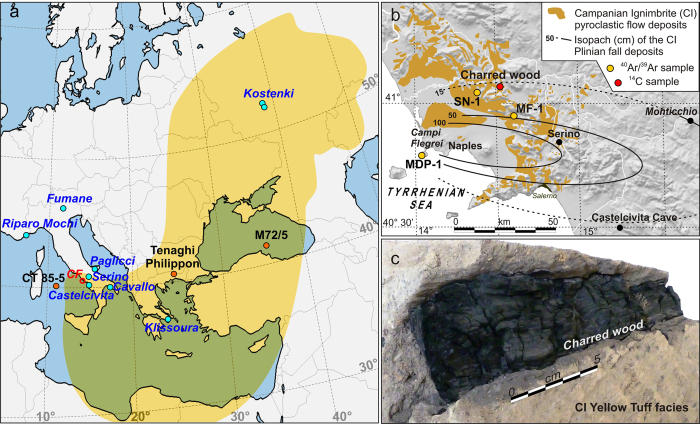
Reference maps and aspect of the charred tree branch embedded in the Campanian Ignimbrite (CI) yellow tuff facies. (**a**) Location of the main palaeoenvironmental records (orange dots) and Palaeolithic sites (blue dots) mentioned in the text. The yellow shaded area is the dispersal areal of the CI tephra within the 0.5 cm isopach of the simulated ash fallout model^3^. CF: Campi Flegrei. (**b**) Geological sketch of the Campanian Ignimbrite (CI) pyroclastic deposits and isopach map of the CI Plinian fall (from ref. 3, 6 and references therein) with the location of the analysed samples of CI deposit for ^40^Ar/^39^Ar (orange dots) and of the ^14^C dated charred tree branch (red dot) embedded in CI pyroclastic showed in panel (**c**) (see also Supplementary Fig. S1). Maps in panel (a and b) were generated using the GIS Open-Source QGIS 2.18 (https://www.qgis.org/it/site/).

Seven pairs of samples were collected from the charred wood embedded in the CI Yellow Tuff (Figs 1c and S1 in Supplementary online material). We applied acid–base–acid (CW#_ABA_) and acid–base oxidation (CW#_ABOX_) pre-treatment procedures (see details in Materials and methods below), and ultimately obtained radiocarbon ages ranging between ~34.6 and ~32.8 ^14^C kyr BP (Table 1). With an exception of two samples (CW5_ABA_ and CW6_ABA_) measured after routine ABA pre-treatment, 12 out of 14 measurements yielded consistent and statistically indistinguishable ages clustering in a narrow temporal interval between ~34.6 and ~34.2 ^14^C kyr BP, independent of the applied ABA or ABOX pre-treatment procedures (Table 1). The two outliers, excluded from the further calculations, may have been affected by contamination that was not successfully removed by the ABA method. However, the two paired samples CW5_ABOX_ and CW6_ABOX_, treated according to the ABOX procedure, provided ages of 34.24 ± 0.30 ^14^C kyr BP and 34.34 ± 0.31 ^14^C kyr BP, i.e., indistinguishable from the other radiocarbon ages obtained by ABA and/or ABOX methods (Table 1). IsoPlot 3.0^34^ was used to calculate the weighted mean and corresponding uncertainty of the 12 retained radiocarbon ages, giving an age of 34.29 ± 0.09 ^14^C kyr BP (1σ). Assuming no pre-aging of the wood, since all ages are in a good agreement this can be considered the statistically most robust radiocarbon age for an organic specimen free of reservoir effect, and virtually coeval to the CI eruption, so far obtained.

### Campanian Ignimbrite ^40^Ar/^39^Ar age

Forty-nine sanidine crystals extracted from three samples of the Campanian Ignimbrite (Fig. 1b) were analyzed using the ^40^Ar/^39^Ar laser incremental heating method (details on sample location and analytical procedure are provided in Materials and methods below). Incremental-heating spectra for the completed experiments are shown in Supplementary Figure S2. While the initial few percent of the gas release frequently yields anomalous ages, either too old or young, most grains yielded apparent-age plateaus, often encompassing >80% of the experiment. With the notable exception of two grains that did not yield plateaus (e.g. Fig. S2n), there is no clear evidence of rising, saddle-shaped or otherwise heterogeneous release patterns that would indicate isotopic discordance. Plateau steps are plotted on inverse isochrons (^36^Ar/^40^Ar vs. ^39^Ar/^40^Ar) in Supplementary Figure S3.

**Figure 2:**
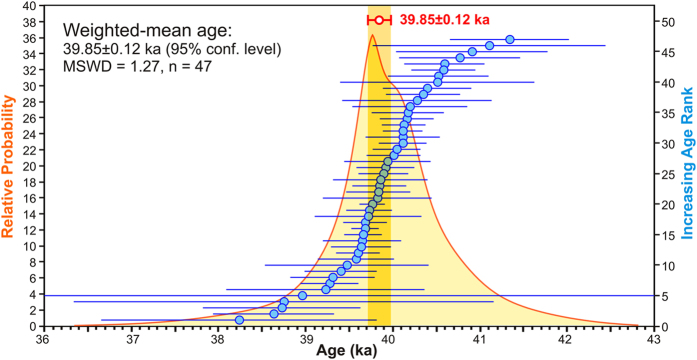
Age-probability density plot of ^40^Ar/^39^Ar single-crystal incremental heating isochron ages for all analysed grains, combined across all samples. The representative weighted-mean age for the CI eruption from this population is 39.85 ± 0.12 ka (95% confidence level, including error in *J*, the neutron-fluence parameter). MSWD, the ‘mean square of weighted deviates’, is a reduced chi-squared statistic that should be approximately unity if the analytical errors are properly estimated and the observed scatter is due to the stated uncertainties. Errors in individual data points (the isochron ages) are shown at 1σ.

Age-probability density spectra and weighted-mean ages of the individual samples are shown in Figure S4. All four samples yield indistinguishable weighted-mean ages ranging from 39.72 to 40.17 ka, with an overall weighted-mean age of the four results of 39.88 ± 0.17 ka (standard error; ±0.28 ka 95% confidence level). However, a statistically more robust treatment of these data can be derived if all experiments are treated as a single aggregate population, an approach justified since all samples are from the same eruption, and individual sample mean ages are mutually indistinguishable at the 95% confidence level. The combined isochron age results are displayed in Fig. 2. The distribution forms a nearly symmetrical, quasi-gaussian curve, with a MSWD = 1.27 (‘mean square of weighted deviates’), and a weighted-mean age of 39.85 ± 0.12 ka (95% confidence level, including the error in *J*, the neutron fluence parameter). This result is combined with prior ^40^Ar/^39^Ar studies of the CI, below.

## Discussion

### Comparing the CI ^40^Ar/^39^Ar ages

Deino *et al*.^35^ reported single-crystal total-fusion and multi-grain resistance furnace ^40^Ar/^39^Ar dating of the CI with an overall age of 37.3 ± 0.8 ka (2σ, recalculated based on the astronomically calibrated age of 28.201 ± 0.046 Ma [2σ] for the Fish Canyon Tuff sanidine^26^ and revised decay constants^36^). This early result, also determined at BGC, is statistically distinct from the new ages reported for the CI given above at the 95% confidence level. We believe an explanation for this discrepancy lies in the numerous improvements in analytical technique in the intervening several decades, including improved monitoring of neutron fluence gradients in irradiation packages, and major improvements in mass resolution, sensitivity, and efficiency afforded by a new generation of multicollector mass spectrometers (e.g. the Nu Instruments *Noblesse* 5-collectors instrument used herein). These improved capabilities permit the adoption of single-grain incremental heating, whereas previously only single-grain total-fusion, or multi-grain step-heating approaches were possible. Additional previous ages for the CI were obtained from both the proximal^2^ and distal^33^ equivalent C-13 (Y-5) layer, which yielded mean ages of 39.85 ± 0.11 ka and 41.4 ± 2.1 ka, respectively (also recalculated for consistency). The result of ref. 2 is virtually identical to the present result, and while that of ref. 33 is older, it nevertheless is statistically indistinguishable from the two more precise values; a weighted-mean of all three ages is 39.85 ± 0.08 ka (2σ; ±0.13 95% confidence level). Because these ages are ultimately tied to astronomically dated Cenozoic sanidine standards from tuffs in Messadit, Morocco; Faneromeni, Crete; and the San Juan Mountains of Colorado via high-precision standard intercalibration studies^26^,^31^,^37^, incorporating the errors in the ages of these monitors increases the overall uncertainty of the CI results by almost negligible amounts. For example, the internal analytical error in the new measurements presented above, ±0.12 ka 2σ, expands to just ± 0.13 ka upon quadratic incorporation of the ±0.1% 2σ uncertainty in the overall age of the Alder Creek sanidine monitor mineral^31^. Further it should be noted that the calculation of Cenozoic ages using Cenozoic monitor standards renders the calculation insensitive to the choice of decay constants used, at the levels of uncertainty involved here. Our final age for the CI, incorporating minor expansion due to uncertainty in the reference age of the astronomical standards, is 39.85 ± 0.14 (95% confidence level).

### Implications for the radiocarbon and calendar time-scales at ~40 ka

**Figure 3:**
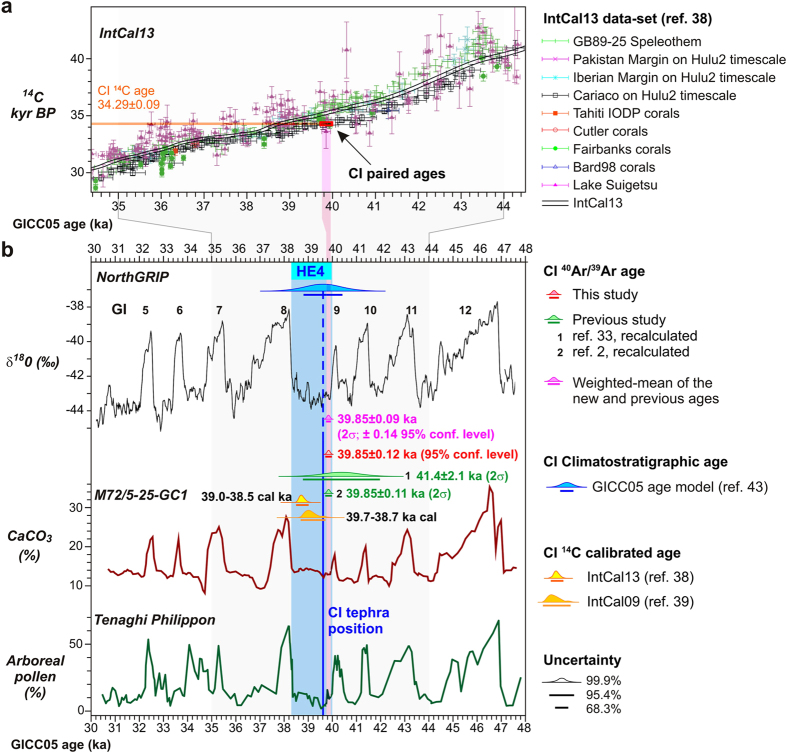
Climatostratigraphic position and ^40^Ar/^39^Ar and ^14^C chronology of the Campanian Ignimbrite (CI) tephra. (**a**) Comparison between the pair of the ^14^C and ^40^Ar/^39^Ar ages of the CI with the IntCal13 curve and related data sets for the 34–44 cal ka BP interval^38^. The pair of ages of the CI falls out of the IntCal13 curve, but fit very well with the Cariaco-Hulu2 Cave and Fairbanks corals data-sets. (**b**) New ^40^Ar/^39^Ar age of the CI compared with previous^2^,^33^ recalculated ^40^Ar/^39^Ar age, the weighted-mean of all new and previous ^40^Ar/^39^Ar ages and the age inferred from CI climatostratigraphic position (blue line). In order to frame the CI climatostratigraphic position within a unique and consistent palaeoclimatic and chronological framework, by using as tie-points the abrupt warming of the onset of the Greenland Interstadial 12 (GI12) to GI5, we aligned the Black Sea (M72/5-25-GC1, 18) and the Tenaghi-Philippon^17^ high-resolution records to the NorthGRIP GICC05 time-scale^42^,^43^. The calibrated radiocarbon ages of the CI, according to the IntCal13^38^ and IntCal09^39^, and the position of the Heinrich Event 4 (HE4) are also shown.

The pair of calendar and ^14^C ages of the CI tephra allows assessment of the calibration of the radiocarbon time-scale in a narrow time-window across CI event (Fig. 3a). For comparison with the paired age of the CI obtained in this study, both IntCal13^38^ and IntCal09^39^ data sets were considered. When using the most recent IntCal13 calibration curve a calibrated radiocarbon age of 38.5–39.0 ka cal BP for the charred wood from the CI tuff is obtained, which is substantially younger than the new ^40^Ar/^39^Ar age of 39.85 ± 0.14 ka. However, calibration with the previous IntCal09 data set results in an age of 39.7–38.7 ka cal BP, which nearly agrees with the new ^40^Ar/^39^Ar age of CI (Fig. 3b). Therefore, our results suggest that IntCal09 provides more accurate radiocarbon calibrations at least for the narrow interval close the CI age and immediately following the Laschamp geomagnetic excursion.

Such an offset of ~1 ka in the calibrated ages has obvious consequences when dealing with detailed correlation between ^14^C dated terrestrial and marine successions with the ice core isotope profile, or with records dated by means other than radiocarbon. To explore the possible reasons for such a difference, we compared our data with the complete data-sets of paired ^14^C and calendar age determinations (Fig. 3a) from which the IntCal13 curve is derived^38^. From this comparison, among other datasets used in IntCal13, a good agreement between our pair of CI ages and the Cariaco on Hulu2 Cave timescale and Fairbanks corals can be noted. Independent of the variability in atmospheric ^14^C content that surrounded the Laschamp Event, our results provide a robust pair of radiocarbon and ^40^Ar/^39^Ar ages obtained for the CI, which can be an important anchor and cross check for future calibration curves.

In this regards, it useful to compare our ^40^Ar/^39^Ar age for the CI with an age that can be inferred from the position of the CI tephra in high-resolution palaeoclimatic records expressing Late Pleistocene sub-millennial climatic oscillations. As shown in previous studies (e.g. refs 19 and 22), the CI tephra falls at the beginning of a stadial event, which is unambiguously correlated to Greenland Stadial 9 (GS9) that, in turn, coincides with Heinrich Event 4 (HE4) in the North Atlantic. This stratigraphic relationship is well expressed in the Tenaghi Philippon pollen^8^,^17^ and in Black Sea geochemical^18^ records (Fig. 3), as well as other palaeoclimatic series (e.g. Tyrrhenian Sea^33^; Monticchio lake^40^; Lesvos Island^16^,^41^). According to the GICC05 age model of the NorthGRIP Greenland isotope profile^42^,^43^, the age corresponding to the CI position in the high-resolution records of the Black Sea and Tenaghi Philippon record is ~39.6 ± 0.8 ka (1σ, 43), which agrees with the age of 39.85 ± 0.14 ka for the CI we have established here. While on one hand this illustrates consistency between the ^40^Ar/^39^Ar geochronological method and the NorthGRIP age model for ref. 43, on the other, the precision of the ^40^Ar/^39^Ar dating for CI is more than one order of magnitude higher then NorthGRIP age model. Therefore, even if the CI is not physically detected in the ice cores of Greenland, through tephra correlation or palaeoclimatic alignments, the new pair of high-precision ^40^Ar/^39^Ar and ^14^C ages can arguably be applied to correlate any records containing either the CI tephra or the HE-4 signal, including NorthGRIP (Fig. 3). The consequent refining of the chronologies of these records has obvious implications for Late Pleistocene time-scale and the radiocarbon calibration near 40 ka.

### The CI and Early Upper Palaeolithic evolutionary processes

**Figure 4:**
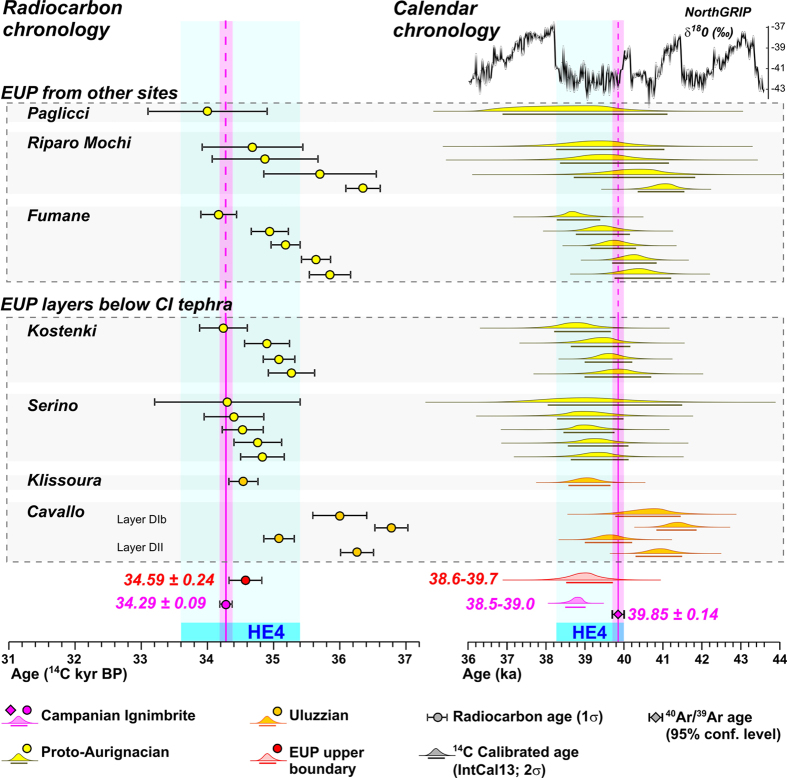
Radiocarbon (left) and calendar (right) chronologies for Campanian Ignimbrite (CI) and Early Upper Palaeolithic (EUP) cultural levels from selected archaeological sites containing (lower panel) and not containing (upper panel) the CI tephra. Independent of the presence of the CI tephra, the upper radiocarbon chronological boundary of the pre-existing EUP cultural industry is close the ^14^C age of the CI. The same chronological relationship is also evident for the calibrated radiocarbon ages of CI and EUP layers (2σ, IntCal13, 38). In contrast, when comparing the EUP radiocarbon calibrated ages (IntCal13, 38) with CI ^40^Ar/^39^Ar dating, an apparent temporal transgression of the EUP upper boundary (38.6–39.7 kyr cal BP) with respect to the CI ^40^Ar/^39^Ar age (39.85 ± 0.14 ka) occurs. The age of the EUP upper boundary (34.59 ± 0.24 ^14^C kyr BP) is the weighted mean of the seven most recent radiocarbon ages from the seven considered sites: Cavallo Cave and Klissoura 1^24^; Serino and Kostenki^22^; Fumane^46^; Paglicci^47^, Riparo Mochi (ref. 48 and references therein, Proto-Aurignacian levels according to ref. 49). The temporal extent of the Heinrich Event 4 (HE4), in both radiocarbon and calendar time-scales, is also shown.

Our new ^14^C age for CI allows us to reassess the timing of the Early Upper Palaeolithic (EUP) bio-cultural evolution in Europe that occurred in very close temporal relationship with CI eruption. Specifically, at several European archaeological sites the CI occurs as a key marker at, or near, the top of EUP cultural horizons of either Proto-Aurignacian or Uluzzian techno-complexes (e.g. refs 6, 7, 8, 19, 22, 23, 24, 25, 26, 27, 28, 29, 30, 31, 32, 33, 34, 35, 36, 37, 38, 39, 40, 41, 42, 43, 44), which have been demonstrated be regionally diachronic cultural phenomena^24^. Specifically, while the Uluzzian persisted in Apulia, southern Italy, and in Greece until ~35–34 ^14^C kyr BP, i.e., until the ^14^C age of the CI eruption (Fig. 4), elsewhere in southern Italy (e.g. Castelcivita, Campania; Fig. 1) this techno-culture was replaced by, or evolved into the Proto-Aurignacian^24^ a few centuries to millennia before the CI eruption. Despite this favourable and unique stratigraphic setting, the lack of reliable radiocarbon dating for the CI has hitherto prevented any direct comparison between the age of the CI and EUP ^14^C chronologies, and thus to palaeoclimatic events (Fig. 3) at the time of the CI eruption.

This issue has been further complicated by the apparent inconsistency of the radiocarbon chronology of the CI itself and of the EUP layers. The first systematic study of the radiocarbon chronology of the archaeological sites containing the CI tephra revealed a substantial anomaly of ^14^C ages of specimens from the Proto-Aurignacian layers resulting in highly scattered and generally much younger chronologies than expected^22^. This appeared consistent with a surprising radiocarbon age reversal observed in Tyrrhenian deep-sea core CT85–5 (Fig. 1), which near the CI tephra fluctuated from *ca*. 35 to *ca*. 17 ^14^C kyr BP^45^. Therefore, in the light of the strict stratigraphic relationship between CI tephra and the Laschamp event/^10^Be peak, a high ^14^C flux was invoked as a possible explanation of this phenomenon^22^. Subsequent improved radiocarbon investigations in archaeological sites containing the CI tephra and techno-cultural horizons strictly correlated to those directly associated with the CI (i.e., Proto-Aurignacian), confirmed the scattered ages observed in the previous EUP ^14^C chronology. However, rather than to a fluctuation of the ^14^C production^22^, the scattering was primarily attributed to contamination and thus to an inadequacy in routine (ABA) sample pre-treatment, as the ABOX pre-treated samples yielded consistent ages^23^,^24^,^46^. In spite of this, two recent radiocarbon ages on marine shells (*Cyclope neritea*) extracted from the layer DI of Cavallo Cave, immediately beneath CI tephra and obtained with a rigorous and more efficient decontamination protocol, yielded again the surprisingly young ages of *ca*. 19.5 ^14^C kyr BP^24^. Therefore, although much of the previous anomalous radiocarbon ages below CI tephra can be certainly related to an inadequate decontaminating pretreatment, the impressive radiocarbon age fluctuation in the CT85–5 record around CI tephra^45^ and some other similar, analytically unjustified, anomalous young ages, remain unexplained, indicating that additional careful investigations across CI interval should be conducted to resolve this issue.

Leaving aside the remnant abnormally young ages and their enigmatic origin, the recent radiocarbon dating of the Proto-Aurignacian or Uluzzian layers immediately beneath CI from Serino, Cavallo Cave, Kostenki and Klissoura 1 archaeological sites (Fig. 1), obtained with ABOx–SC (charcoal) or CarDS (shell) protocol^24^,^25^, yielded ages statistically indistinguishable or barely older than the ^14^C age (either calibrated or uncalibrated) of the CI eruption (Fig. 4). This adds new chronological evidence to the debate about the possible direct or indirect connection between the CI super-eruption and the EUP bio-cultural evolution (e.g. refs 3, 7, 8, 10, 19, 20, 21, 22). Specifically, our data reveal that no appreciable temporal hiatus separates the latest EUP occupation layers from the CI deposition, thus confirming the strict temporal relationship between CI eruption and site abandonment and/or cultural change, postulated on the basis of the stratigraphic evidence alone^19^.

The same temporal coincidence is also found at other Italian EUP sites that do not contain the CI tephra. The most recent, and, in some cases, improved ABOx–SC radiocarbon chronology of the Proto-Aurignacian levels from the Italian sites of Fumane^46^, Paglicci^47^ and Riparo Mochi (ref. 48 and references therein, Proto-Aurignacian levels according to ref. 49) reveal that the final stages of this techno-culture approach the ^14^C age of either CI eruption or of the cultural levels from the CI-sites sealed by the tephra (Fig. 4). In other words, the evidence indicates that: (*i*) the Proto-Aurignacian or Uluzzian industries either stratigraphically or chronologically never cross the CI eruption event, and (*ii*) the CI age coincides, within uncertainty, with the upper chronological boundary of the EUP techno-complexes (Fig. 4). Indeed, the weighted mean of the radiocarbon age of the latest Uluzzian or Proto-Aurignacian layers, from sites either containing or not containing the CI tephra, is 34.59 ± 0.24 ^14^C kyr BP, i.e., statistically indistinguishable from the CI ^14^C age (34.29 ± 0.09 kyr BP). Although a mere coincidence of two independent events-processes cannot be completely excluded, we are inclined to consider the strict matching between CI age and the end of the Uluzzian/Proto-Aurignacian cultures as a significant chronological evidence supporting the notion of impact of the CI eruption (19; *contra* 8), according to which the apparent abrupt end of the lithic traditions preceding the CI, the interruption of the human occupation, and the following lithic industry evolution can be seen as adaptive and accelerated responses to the drastic and rapid climatic-environmental modifications induced by the CI-HE4 combined event.

It is worth noting that when comparing the EUP radiocarbon calibrated ages (IntCal13, 38) directly with the CI ^40^Ar/^39^Ar dating, the latest EUP occurrences and its upper chronological limit (38.6–39.7 kyr cal BP) appear distinctly younger than CI ^40^Ar/^39^Ar age (39.85 ± 0.14 ka) (Fig. 4). A similar erroneous conclusion results from the comparison of the radiocarbon calibrated ages of the EUP with the Greenland record, according to which the Uluzzian/Proto-Aurignacian cultures seem to be declining during the HE4 events, rather than before HE4, as our data suggest (Fig. 4). These are practical examples of how, especially when dealing with refined chronological issues, comparing events/processes dated with different, imprecisely intercalibrated methods may be hazardous and lead to incorrect historical reconstructions.

### Summary and concluding remarks

The consolidated ^40^Ar/^39^Ar age of the CI at 39.85 ± 0.14 ka (95% confidence level) provides a high-precision chronostratigraphic tie-point for this widespread super-marker tephra that, through direct tephra correlation or palaeoclimatic alignment can be transferred and applied to a number of sedimentary successions for refining the chronology of a series of natural and cultural occurrences. The paired atmospheric radiocarbon and ^40^Ar/^39^Ar dating of the CI eruption provide the first reliable intercalibrated ^14^C-^40^Ar/^39^Ar age for this super-tephra, which represents a robust data for refining the Late Pleistocene time-scale and the radiocarbon calibration curve. While the new ^40^Ar/^39^Ar age for CI is in good agreement with both NorthGRIP GICC05 time-scale and to some extent with the IntCal09-calibrated age of the CI, IntCal13 yields systematically younger calibrated ages. Specifically, for samples coeval or slightly older than CI, IntCal13 provide calibrated ages that are nearly 1 ka younger than CI ^40^Ar/^39^Ar and NorthGRIP GICC05 ages. Regardless of any assessment and judgment on the accuracy of both ^40^Ar/^39^Ar and radiocarbon calibrated age of CI, this highlights the need for accurate inter-calibration when evaluating high resolution temporal relationships, and the chronological order of events evaluated by different dating methods.

As far as the methodological aspects are concerned, the multiple approaches used for dating the charred wood found in the CI tuff showed that standard ABA and the improved ABOX treatments were successfully applied to five of seven (~71%) and to all (100%) samples, respectively.

Finally, although we are aware of the current incompleteness of the EUP radiocarbon chronology, and of the limits inherent the assessment of EUP stratigraphic integrity, our data suggest that in a wide region extending from Italy to eastern Europe the CI eruption marked an abrupt end to the millennial-long Uluzzian or Proto-Aurignacian techno-cultures. Compelling chronological evidence now supports a scenario that emphasizes the evolutionary role of the combined CI-HE4 event.

## Materials and Methods

### Stratigraphic and volcanological setting of the charred wood branch

The CI deposits containing the charred tree branch analysed here was exposed in a quarry close to the town of Dugenta (Fig. 1b; 41°07′16.79″ N, 14°26′32.55″ E). Here, the CI exhibits a typical eruptive succession of basal Plinian pumice fallout overlain by ~15 m of pyroclastic flow deposit. The flow is poorly welded to partially welded grey to dark grey tuff, grading upward into zeolitised yellow tuff. Our sample was found embedded in the yellow tuff facies (Fig. 1c), and likely represents the branch of a tree entrained by the pyroclastic flow during runout. The charred branch is ~25 cm long and has a maximum diameter of ~8 cm, along which at least 15 tree rings can be observed (Fig. S1). Fragments of branch and trunk embedded in the CI yellow tuff in other areas of the NE sector of the Campanian Plain (e.g. Tufino quarries; ref. 50) indicates that the emplacement of the CI pyroclastic flow led to the uprooting and burning of woody vegetation living in the area at the time of the eruption. This implies that, ignoring the negligible pre-eruption age of the tree, the sample analysed here is coeval with the eruption.

### ^14^C dating of the charred wood branch

As a part of an ongoing study at ETH laboratory, all samples that are older than 20 ka are prepared using two preparation methods: ‘ABA’ and ‘ABOX’. The first results obtained on charcoal from Chauvet Cave (32 ka ^14^C BP) have demonstrated the compatibility of the methods^51^. The charred wood from CI tuff provided an excellent material for such a comparison. This charred wood was split and both subsamples treated first with Acid Base Acid (ABA) treatment to remove contamination with foreign carbon^52^. In this treatment wood was submerged in a weak acid bath (0.5 M HCl) at 60 °C for 12 hrs, by which means carbonates are removed from the sample. Following multiple washes in distilled water, an alkali step was applied. A weak base (0.1 M NaOH) at 60 °C for 12 hrs, which removes humic acids that might contaminate wood, was followed with washes to neutral pH. The last step of acid wash was applied to finally remove any atmospheric carbon that could be bond to charcoal during the base step. At this point additional treatment has only been applied to the second sub-sample (ABOX). Following the procedure of Bird *et al*.^53^, the oxidizing step 0.1 M K_2_Cr_2_O_7_ in a 2 M solution H_2_SO_4_ was applied for 30 min at 60 °C. All the steps in the treatment were followed by washing the sample with pure water (Mili-Q^®^) to neutral pH.

The dried samples (1 mg of C) were then combusted in Elemental Analyzer and subsequently graphitized, pressed into the targets and analyzed with accelerator mass spectrometer AMS^54^ along with blank and standard samples. Blank samples made of ^14^C free phthalic anhydride were prepared to monitor contamination during the graphitization process.

Conventional ^14^C ages were calculated using the method of Stuiver and Polach^55^. These ages include correction for fractionation based on on-line measurements of δ^13^C on graphite. The 1-sigma error includes counting uncertainty as well as the scatter of standards and blanks.

^14^C ages were calibrated using OxCal 4.1 program^56^ and IntCal09 and IntCal13 calibration data sets^38^,^39^.

### ^39^Ar/^40^Ar dating of the Campanian Ignimbrite

Sanidine phenocrysts from three samples of the Campanian Ignimbrite were analyzed on a grain-by-grain basis using the ^40^Ar/^39^Ar laser incremental heating method. Sample SN-1 was separated from pumice collected from non-welded ignimbrite exposed in a large commercial quarry in the vicinity of San Nicola la Strada near Maddaloni (Fig. 1b; 41°02′33.22″ N, 14°21′04.29″ E). MDP-1 is pumice from a ~20 m thick poorly bedded proximal Campanian Ignimbrite (‘Breccia Museo’) exposed on Monte di Procida Island at the Marina di Vita Fumo (Fig. 1b; 40°47′24.11″ N, 14°03′05.45″ E). MF-1 is pumice from unwelded ignimbrite collected along the main road through Monte Forte near Avellino, outside the town of Baiano (Fig. 1b; 41°07′16.79″ N; 14°26′32.55″ E).

Sanidine phenocrysts were separated by gentle crushing and handpicked followed by dilute HF and distilled water washes. Final selection of grains was performed under water, to permit inclusion-free material to be retrieved. Finished separates were loaded into aluminum sample holders, which were then stacked, wrapped in aluminum foil, and sealed in quartz vials for irradiation in the Cd-lined in-core CLICIT position of the Oregon State University TRIGA reactor.

Samples SN-1, MDP-1, and MF-1 were irradiated for 30 minutes (irradiation #419), while SN-1r, a second aliquot of SN-1, was irradiated for 5 minutes (#420). The neutron fluence monitor mineral for both irradiations was sanidine from the Alder Creek Rhyolite of California (age = 1.1848 ± 0.006 Ma;^31^). Reactor-induced isotopic production ratios for this irradiation were: (^36^Ar/^37^Ar)_Ca_ = 2.65 ± 0.02 × 10^−4^, (^38^Ar/^37^Ar)_Ca_ = 1.96 ± 0.08 × 10^−5^, (^39^Ar/^37^Ar)_Ca_ = 6.95 ± 0.09 × 10^−4^, (^37^Ar/^39^Ar) _K_ = 2.24 ± 0.16 × 10^−4^, (^38^Ar/^39^Ar)_K_ = 1.220 ± 0.003 × 10^−2^, (^40^Ar/^39^Ar) _K_ = 2.5 ± 0.9 × 10^−4^. Atmospheric ^40^Ar/^36^Ar = 298.56 ± 0.31^57^ and decay constants were according to ref. 36.

Following irradiation, 13, 14, 13, and 9 grains of MF-1, MDP-1, SN-1, and SN-1r, respectively, were analysed by the incremental heating ^40^Ar/^39^Ar method (Supplementary data-set DS1), using a *Noblesse* multi-collector noble gas mass spectrometer. ^40^Ar, ^37^Ar, and ^36^Ar were measured simultaneously on separate ion counters, while peak hopping was used to measure ^39^Ar on the same ion counter as that employed for ^40^Ar. Further details of the multicollection protocol is provided in ref. 58. Complete degassing of the grains was achieved in 10–28 steps. ^40^Ar/^39^Ar analytical data are provided in Supplementary data-set DS1.

## Additional Information

**How to cite this article:** Giaccio, B. *et al*. High-precision ^14^C and ^40^Ar/^39^Ar dating of the Campanian Ignimbrite (Y-5) reconciles the time-scales of climatic-cultural processes at 40 ka. *Sci. Rep.* **7**, 45940; doi: 10.1038/srep45940 (2017).

**Publisher's note:** Springer Nature remains neutral with regard to jurisdictional claims in published maps and institutional affiliations.

## Supplementary Material

Supplementary Figures

Supplementary Dataset

## Figures and Tables

**Table 1:** Results of AMS ^14^C analysis for the seven samples extracted from the charred wood (CW) embedded in the Campanian Ignimbrite Yellow Tuff (Fig. 1) performed after both ABA and ABOx–SC pre-treatment procedures.

Sample	Pre-treatment	^14^C age^1^ (yrs BP^2^)	±1σ (yrs)	δ^13^C (‰)^3^	±1σ (‰)
CW1	ABA	34564	339	−25.7	1.1
	ABOx	34232	306	−22.0	1.1
CW2	ABA	34168	329	−26.1	1.1
	ABOx	34352	313	−23.7	1.1
CW3	ABA	34232	329	−23.7	1.1
	ABOx	34362	308	−20.4	1.1
CW4	ABA	34283	330	−24.2	1.1
	ABOx	34188	307	−24.3	1.1
CW5	ABA	*32825*	*279*	−25.2	1.1
	ABOx	34244	306	−22.6	1.1
CW6	ABA	*33751*	*315*	−26.0	1.1
	ABOx	34343	312	−23.9	1.1
CW7	ABA	34386	337	−25.6	1.1
	ABOx	34183	305	−21.7	1.1
**Weighted mean**^4^		**34290**	**90**		

^1^Delta ^13^C corrected radiocarbon age^53^ based on concentration of ^14^C measured in sample.

^2^Before Present = before 1950 AD.

^3^δ^13^C is a value measured on graphite and might include additional fractionation.

^4^Not including the CW5_ABA_ and CW6_ABA_ ages (in italic).

All samples 1 mg of C and C/N ratio between 190 and 285.

## References

[b1] Lowe D. J. Tephrochronology and its application: A review. Quat. Geochron. 6, 107–153 (2011).

[b2] De Vivo B. . New constraints on the pyroclastic eruptive history of the Campanian Volcanic Plain (Italy). Contrib. Mineral Petrol. 73, 47–65 (2001).

[b3] Costa A. . Quantifying volcanic ash dispersal and impact of the Campanian Ignimbrite super-eruption. Geophys. Res. Lett. 39(10) (2012).

[b4] Thunnel R., Federman A., Sparks S. & Williams D. The age, origin, and volcanological significance of the Y-5 ash layer in the Mediterranean. Quat. Res. 12, 241–253 (1979).

[b5] Pyle D. M. . Wide dispersal and deposition of distal tephra during the Pleistocene ‘Campanian Ignimbrite/Y5′ eruption, Italy. Quat. Sci. Rev. 25, 2713–2728 (2006).

[b6] Giaccio B. . The Campanian Ignimbrite and Codola tephra layers: Two temporal/stratigraphic markers for the Early Upper Palaeolithic in southern Italy and eastern Europe. J. Volcanol. Geotherm. Res. 177, 208–226 (2008).

[b7] Fedele F. G., Giaccio B., Isaia R. & Orsi G. Ecosystem impact of the Campanian Ignimbrite eruption in Late Pleistocene Europe. Quat. Res. 57, 420–424 (2002).

[b8] Lowe J. . Volcanic ash layers illuminate the resilience of neanderthals and early modern humans to natural hazards. Proc. Natl. Acad. Sci. 109, 13532–13537 (2012). 22826222 10.1073/pnas.1204579109 PMC3427068

[b9] Morley M. W. & Woodward J. C. The Campanian Ignimbrite (Y5) tephra at Crvena Stijena Rockshelter, Montenegro. Quat. Res. 75, 683–696 (2011).

[b10] Fitzsimmons K. E., Hambach U., Veres D. & Iovita R. The Campanian Ignimbrite eruption: New data on volcanic ash dispersal and its potential impact on human evolution. PLOS ONE 8(6) (2013). 10.1371/journal.pone.0065839 PMC3684589 23799050

[b11] Douka K. . The chronostratigraphy of the Haua Fteah Cave (Cyrenaica, northeast Libya). J. Hum. Evol. 66, 39–63 (2014). 24331954 10.1016/j.jhevol.2013.10.001

[b12] Smith V. C., Isaia R., Engwell S. L. & Albert P. G. Tephra dispersal during the Campanian Ignimbrite (Italy) eruption: implications for ultra-distal ash transport during the large caldera-forming eruption. Bull. Volcanol. 78, 45 (2016).

[b13] Tomlinson E. L. . Geochemistry of the Phlegraean Fields (Italy) proximal sources for major Mediterranean tephras: Implications for the dispersal of Plinian and co-ignimbritic components of explosive eruptions. Geochim. Cosmochim. Acta 93, 102–128 (2012).

[b14] Scarpati C. & Perrotta A. Stratigraphy and physical parameters of the Plinian phase of the Campanian Ignimbrite eruption. Bull. Geol. Soc. Am. 128, 1147–1159 (2016).

[b15] Engwell S. L., Sparks R. S. J. & Carey S. Physical characteristics of tephra layers in the deep sea realm: the Campanian Ignimbrite eruption. Geol. Soc. London Spec. Publ. 398, SP398–7 (2014).

[b16] Margari V., Pyle D. M., Bryant C. & Gibbard P. L. Mediterranean tephra stratigraphy revisited: Results from a long terrestrial sequence on Lesvos Island, Greece. J. Volcanol. Geotherm. Res. 163, 34–54 (2007).

[b17] Müller U. C. . The role of climate in the spread of modern humans into Europe. Quat. Sci. Rev. 30, 273–279 (2011).

[b18] Nowaczyk N. R., Arz H. W., Frank U., Kind J. & Plessen B. Dynamics of the Laschamp geomagnetic excursion from black sea sediments. Earth Planet Sci. Lett. 351–352, 54–69 (2012).

[b19] Fedele F. G., Giaccio B. & Hajdas I. Timescales and cultural process at 40,000 BP in the light of the Campanian Ignimbrite eruption, western Eurasia. J. Hum. Evol. 55, 834–857 (2008). 18922561 10.1016/j.jhevol.2008.08.012

[b20] Golovanova L. V. . Significance of ecological factors in the Middle to Upper Paleolithic transition. Curr. Anthropol. 51, 655–691 (2010).

[b21] Black B. A., Neely R. R. & Manga M. Campanian Ignimbrite volcanism, climate, and the final decline of the Neanderthals. Geology 43, 411–414 (2015).

[b22] Giaccio B., Hajdas. I., Peresani M., Fedele F. G. & Isaia R. The Campanian Ignimbrite tephra and its relevance for the timing of the Middle to Apper Palaeolithic shift in *When Neanderthals and Modern* Humans Met (ed. Conard N. J.) 343–375 (Kerns Verlag, Tübingen, Germany, 2006).

[b23] Wood R. E. . Testing the ABOx-SC method: Dating known-age charcoals associated with the Campanian Ignimbrite. Quat. Geochron. 9, 16–26 (2012).

[b24] Douka K. . On the chronology of the Uluzzian. J. Hum. Evol. 68, 1–13 (2014). 24513033 10.1016/j.jhevol.2013.12.007

[b25] Ronchitelli A., Benazzi S., Boscato P., Douka K. & Moroni A. Comments on “human-climate interaction during the early Upper Paleolithic: Testing the hypothesis of an adaptive shift between the Proto-Aurignacian and the Early Aurignacian” by William E. Banks, Francesco d’Errico, Ioão Zilhão. J. Hum. Evol. 72, 107–111 (2014). 10.1016/j.jhevol.2013.12.010 24529865

[b26] Kuiper K. F. . Synchronizing rock clocks of Earth history. Science 320, 500–504 (2008). 18436783 10.1126/science.1154339

[b27] Rivera T. A., Storey M., Zeeden C., Hilgen F. J. & Kuiper K. A. Refined astronomically calibrated ^40^Ar/^39^Ar age for Fish Canyon sanidine. Earth Planet Sci. Lett. 311, 420–426 (2011).

[b28] Renne P. R., Balco G., Ludwig K. R., Mundil R. & Min K. Response to the comment by W.H. Schwarz *et al*. on “Joint determination of ^40^K decay constants and ^40^Ar*/ ^40^K for the fish canyon sanidine standard, and improved accuracy for ^40^Ar/^39^Ar geochronology” by P.R. Penne *et al*. (2010). Geochim. Cosmochim. Acta 75, 5097–5100 (2011).

[b29] Jicha B. R., Singer B. S. & Sobol P. Re-evaluation of the ages of ^40^Ar/^39^Ar sanidine standards and supereruptions in the western U.S. using a Noblesse multi-collector mass spectrometer. Chemical Geology 431, 54–66 (2016).

[b30] Phillips D. & Matchan E. L. Ultra-high precision ^40^Ar/^39^Ar ages for Fish Canyon Tuff and Alder Creek Rhyolite sanidine: New dating standards required. Geochim. Cosmochim. Acta 121, 229–239 (2013).

[b31] Niespolo E. M., Rutte D., Deino A. L. & Renne P. R. Intercalibration and age of the Alder Creek sanidine ^40^Ar/^39^Ar standard. Quat. Geochron. doi: 10.1016/ j.quageo.2016.09.004 (2016).

[b32] Fleck R. J. & Calvert A. T. Intercalibration of ^40^Ar/^39^Ar mineral standards with Bodie Hills Sanidine. Geological Society of America abstract 238, 4 (2016).

[b33] Ton-That T., Singer B. & Paterne M. ^40^Ar/^39^Ar dating of latest Pleistocene (41 ka) marine tephra in the Mediterranean Sea: Implications for global climate records. Earth Planet Sci. Lett. 184, 645–58 (2001).

[b34] Ludwig K. R. Isoplot 3.0-a Geochronological Toolkit for Microsoft Excel. In Special Publication No. 4, Berkeley Geochronology Center: Berkeley, Calif (2001).

[b35] Deino A., Curtis G. H., Southon J., Terrasi F., Campajola L. & Orsi G. ^14^C and ^40^Ar/^39^Ar dating of the Campanian Ignimbrite, Phlegrean Fields, Italy. ICOG 8, USGS Circ. 1107, 77 (1994).

[b36] Min K., Mundil R., Renne P. R. & Ludwig K. R. A test for systematic errors in ^40^Ar/^39^Ar geochronology through comparison with U/Pb analysis of a 1.1-Ga rhyolite. Geochim. Cosmochim. Acta 64, 73–98 (2000).

[b37] Renne P. R. . Intercalibration of standards, absolute ages and uncertainties in ^40^Ar/^39^Ar dating. Chem. Geol. 145, 117–52 (1998).

[b38] Reimer P. J. . IntCal13 and Marine13 radiocarbon age calibration curves 0–50,000 years cal BP. Radiocarbon 55, 1869–1887 (2013).

[b39] Reimer P. J. . IntCal09 and Marine09 radiocarbon age calibration curves, 0–50,000 years CAL BP. Radiocarbon 51, 1111–1150 (2009).

[b40] Watts W. A., Allen J. R. M. & Huntley B. Palaeoecology of three interstadial events during oxygen-isotope stages 3 and 4: A lacustrine record from lago grande di Monticchio, southern Italy. Palaeogeogr. Palaeoclimatol. Palaeoecol. 155, 83–93 (2000).

[b41] Margari V., Gibbard P. L., Bryant C. L. & Tzedakis P. C. Character of vegetational and environmental changes in southern Europe during the Last Glacial period; evidence from Lesvos Island, Greece. Quat. Sci. Rev. 28, 1317–39 (2009).

[b42] Svensson A. . The Greenland ice core chronology 2005, 15–42 ka. Part 2: Comparison to other records. Quat. Sci. Rev. 25, 3258–67 (2006).

[b43] Svensson A. . A 60000 year greenland stratigraphic ice core chronology. Climate of the Past 4, 47–57 (2008).

[b44] Hoffecker J. F. . From the bay of Naples to the River Don: The Campanian Ignimbrite eruption and the Middle to Upper Paleolithic transition in eastern Europe. J. Hum. Evol. 55, 858–70 (2008). 18937961 10.1016/j.jhevol.2008.08.018

[b45] Hajdas I. . Anomalous radiocarbon ages found in Campanian Ignimbrite deposit of the Mediterranean deep-sea core CT85-5. Radiocarbon 53, 575–583 (2011).

[b46] Higham T. . Problems with radiocarbon dating the middle to Upper Palaeolithic transition in Italy. Quat. Sci. Rev. 28, 1257–1267 (2009).

[b47] Palma di Cesnola A. La séquence de la grotte Paglicci (Mont Gargano) dans le cadre du Leptolithique de l’Italie méridionale. In Les faciès leptolithiques du nord-ouest méditerranéen: milieux naturels et culturels (ed. Sacchi D.) 185–194 (Préhistorique de France, Carcassonne, 26–30 September 1994, 1999).

[b48] Douka K., Grimaldi S., Boschian G., del Lucchese A. & Higham T. F. G. A new chronostratigraphic framework for the Upper Palaeolithic of Riparo Mochi (italy). J. Hum. Evol. 62, 286–299 (2012). 22189428 10.1016/j.jhevol.2011.11.009

[b49] d’Errico F. & Banks W. Tephra studies and the reconstruction of Middle-to-Upper Paleolithic cultural trajectories. *Quat. Sci. Rev.* 118, 182–103 (2015).

[b50] Alessio M. F., Bella F., Improta S., Belluomini G., Cortesi C. & Turi B. University of Rome Carbon-14 Dates X. Radiocarbon 15, 165–178 (1973).

[b51] Quiles A. . Second Radiocarbon Intercomparison Program for the Chauvet-Pont d’Arc Cave, Ardèche, France. Radiocarbon 56, 833–850 (2014).

[b52] Hajdas I. The Radiocarbon dating method and its applications in Quaternary studies. Quaternary Science Journal - Eiszeitalter und Gegenwart 57, 2–24 (2008).

[b53] Bird M. I. . Radiocarbon dating of “old” charcoal using a wet oxidation, stepped-combustion procedure. Radiocarbon 41, 127–140 (1999).

[b54] Synal H. A., Stocker M. & Suter M. MICADAS: A new compact radiocarbon AMS system. Nucl. Instrum. Meth. B 259, 7–13 (2007).

[b55] Stuiver M. & Polach H. A. Reporting of C-14 Data - Discussion. Radiocarbon 19, 355–363 (1977).

[b56] Bronk Ramsey C. OxCal 4.2 manual https://c14.arch.ox.ac.uk/oxcalhelp/hlp_contents.html (2010).

[b57] Lee J.-Y. . A redetermination of the isotopic abundances of atmospheric Ar. Geochim. Cosmochim. Acta 70, 4507–4512 (2006).

[b58] Di Maggio E. N. . Late Pliocene fossiliferous sedimentary record and the environmental context of early Homo from Afar, Ethiopia. Science 347, 1355–1359 (2015). 25739409 10.1126/science.aaa1415

