# Supplementary material for: High-precision 14C and 40Ar/39Ar dating of the Campanian Ignimbrite (Y-5) reconciles the time-scales of climatic-cultural processes at 40 ka
Source: Sci Rep. 2017 Apr 6;7:45940. doi: 10.1038/srep45940 (PMC5382912; doi:10.1038/srep45940)
Supplement: Supplementary Figures [file srep45940-s1.pdf]

High-precision ^{14}C and ^{40}Ar/^{39}Ar dating of the Campanian Ignimbrite (Y-5) reconciles the time-scales of climatic-cultural processes at 40 ka

Biagio Giaccio[1]*, Irka Hajdas[2], Roberto Isaia[3], Alan Deino[4], Sebastien Nomade[5]

[1] CNR - Istituto di Geologia Ambientale e Geoingegneria (IGAG), Via Salaria km 29.300, 00015 Monterotondo, Rome, Italy.
[2] Laboratory of Ion Beam Physics, ETH, Otto-Stern-Weg 5, 8093 Zürich, Switzerland.
[3] Istituto Nazionale di Geofisica e Vulcanologia (INGV), Sezione di Napoli, Osservatorio Vesuviano, Via Diocleziano 328, 80124 Naples, Italy.
[4] Berkeley Geochronology Center, 2455 Ridge Road, Berkeley, California 94709, USA.
[5] Laboratoire des Sciences du Climat et de L'Environnement (IPSL-CEA-CNRS-UVSQ) et Université de Paris-Saclay, Domaine du CNRS Bât. 12, Avenue de la Terrasse, 91198 Gif sur Yvette, France.

*Corresponding author: biagio.giaccio@cnr.it

Supplementary Figures

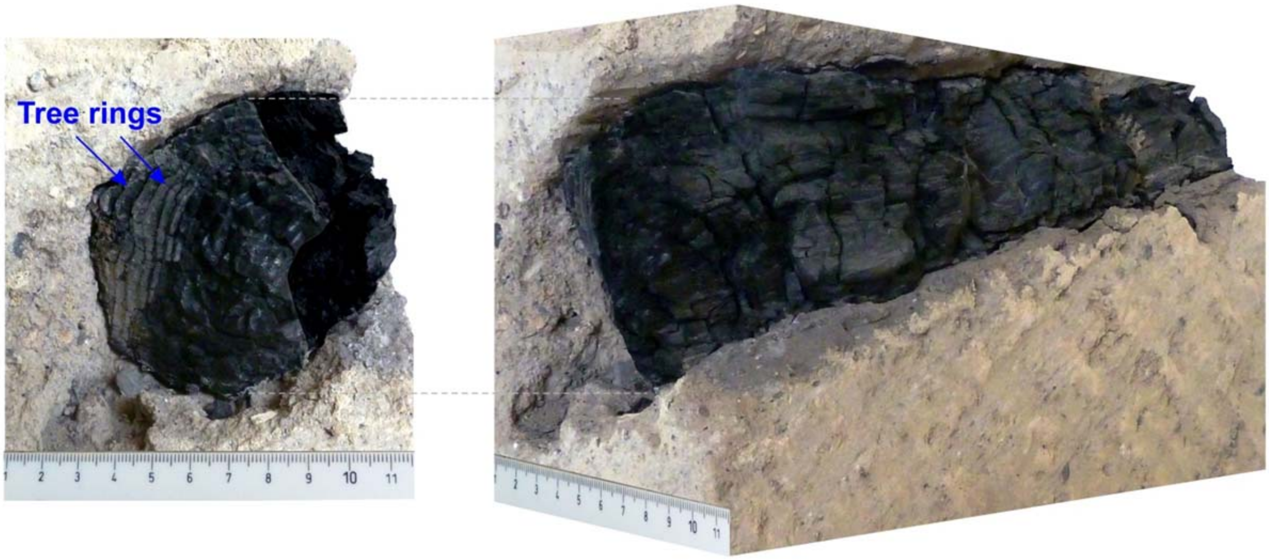

Figure S1. Aspect of the charred wood embedded in Campanian Ignimbrite Yellow Tuff facies.

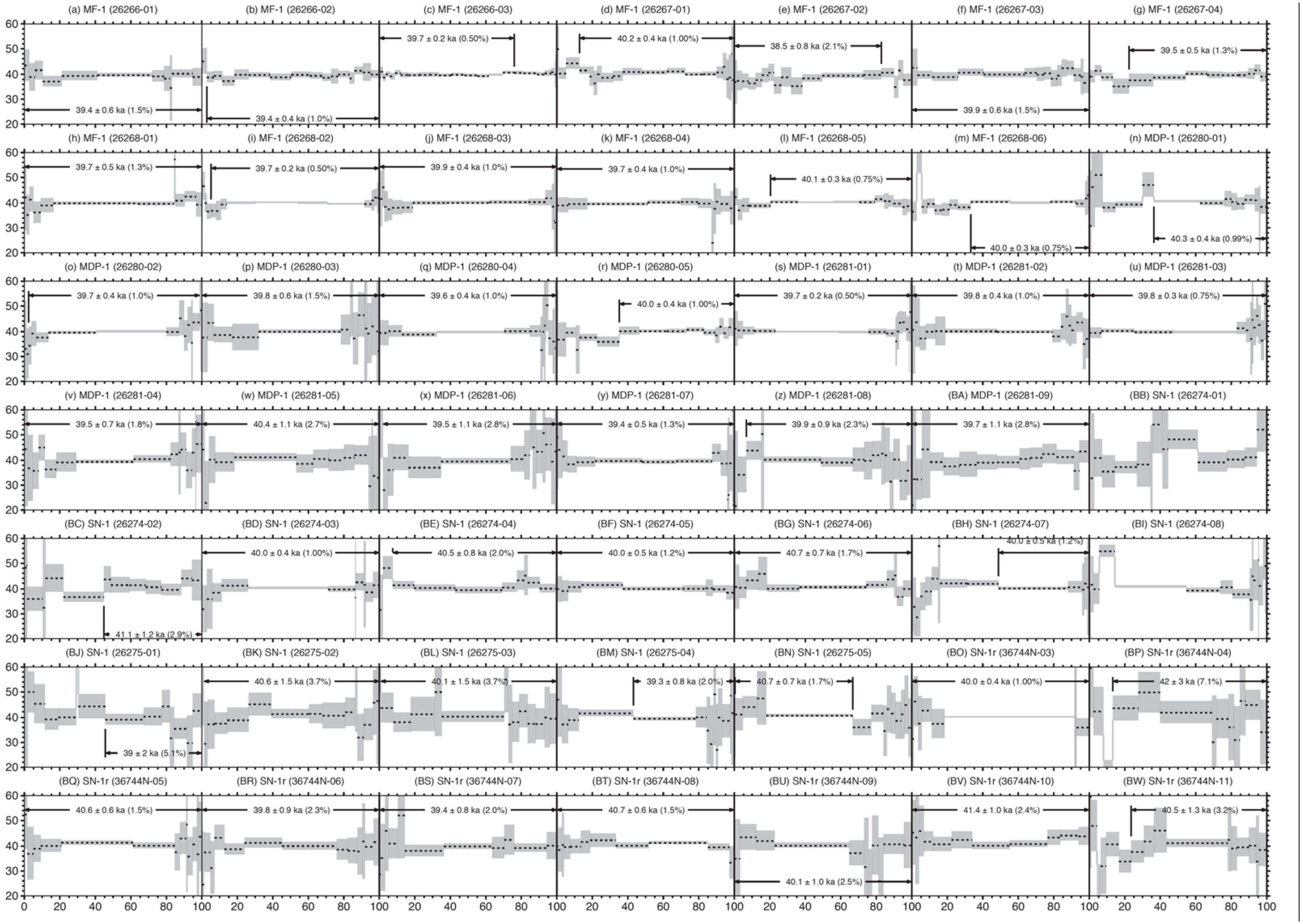

Figure S2. Incremental heating spectra for the sanidine single-crystal $^{40}Ar/^{39}Ar$ experiments.

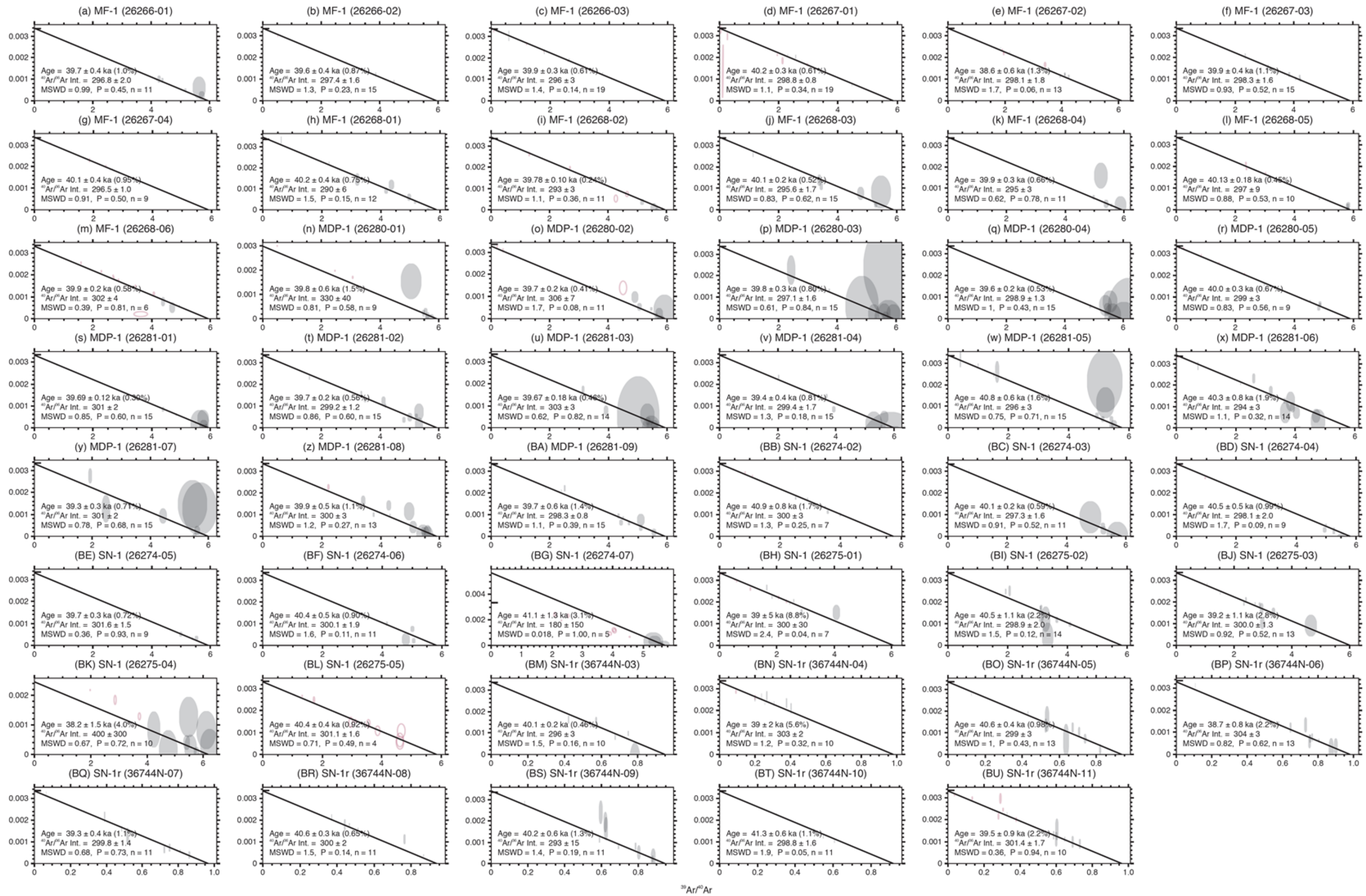

Figure S3. ‘Inverse isochron’ plots of plateau steps (each step-heating experiment plotted separately). The ages derived from the individual crystal isochrons are carried forward to the final age evaluation.

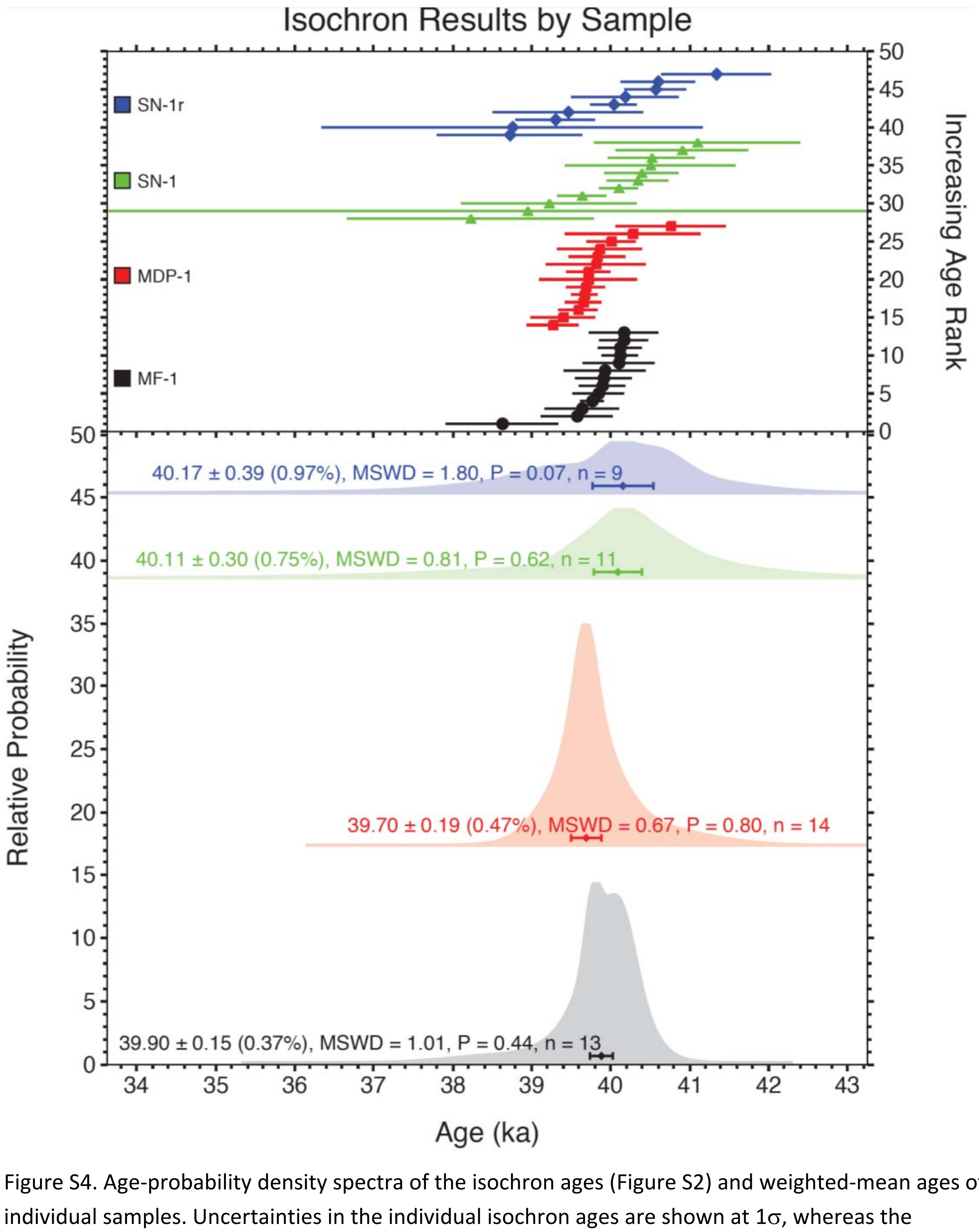

Figure S4. Age-probability density spectra of the isochron ages (Figure S2) and weighted-mean ages of the individual samples. Uncertainties in the individual isochron ages are shown at 1σ, whereas the uncertainties in the weighted means are expressed as the standard error, expanded by root(MSWD) where MSWD>1. It can be seen from the uncertainties of the individual analyses that SN-1 and SN-1r $^{40}Ar/^{39}Ar$ experiments give greater uncertainty on average than MF-1 and MDP-1 principally because SN-1 yields a lower percentage of radiogenic ^{40}Ar (%^{40}Ar *) relative to total ^{40}Ar, hence require a greater atmospheric argon contamination correction. Additionally, SN-1r grains were smaller, about one-tenth the volume of the other three comparably sized samples. Whereas SN-1r required less correction for atmospheric argon contamination relative to SN-1, the smaller grain size resulted in less favourable spectrometer counting statistics, leading to similar mean age uncertainties.
